# Proteomic Analysis of the Follicular Fluid of Tianzhu White Yak during Diestrus

**DOI:** 10.3390/ijms15034481

**Published:** 2014-03-13

**Authors:** Jinzhong Tao, Guoshun Zhao, Xingxu Zhao, Fadi Li, Xiaohu Wu, Junjie Hu, Yong Zhang

**Affiliations:** 1 Agricultural College, Ningxia University, Yinchuan 750021, China; E-Mail: tao_jz@nxu.edu.cn; 2 College of Veterinary Medicine, Gansu Agricultural University, Lanzhou 730070, China; E-Mails: zhaogs111@163.com (G.Z.); wx.258.h@st.gsau.edu.cn (X.W.); hujj@gsau.edu.cn (J.H.); zhychy@163.com (Y.Z.); 3 College of Animal Science and Technology, Gansu Agricultural University, Lanzhou 730070, China

**Keywords:** Tianzhu white yak, follicular fluid, proteomic analysis, two-dimensional gel electrophoresis, diestrus

## Abstract

The aim of this study was to identify differentially expressed proteins in the follicular fluid of Tianzhu white yak during diestrus. Follicles obtained from female yak were divided into four groups according to their diameter: 0–2, 2–4, 4–6 mm, and greater than 6 mm. The follicular fluid was directly aspirated from the follicles and mixed according to follicular size, and two-dimensional gel electrophoresis was carried out on the crude follicular fluid samples. Thirty-four differentially expressed spots were generated from these four sizes of follicles. Fourteen of these spots were analyzed by MALDI-TOF/TOF-MS and identified as: AS3MT, VDP, ANKRD6, C10orf107 protein, MRP4, MAPKAP1, AGO3, profilin-β-actin, SPT2 homolog, AGP, AR, RNF20, obscurin-like-1, and one unnamed protein. These proteins were first reported in follicular fluid, in addition to VDP and AGP. Based on existing knowledge of their function and patterns of expression, we hypothesize that most of these differentially expressed proteins play a role in ovarian follicular growth and development, dominant follicle selection, or follicular atresia and development of oocytes; however, the function of the other differentially expressed proteins in reproduction remains ambiguous.

## 1. Introduction

The Tianzhu white yak (*Poephagus grunniens*) inhabits cold prairies above 3000 m, and is a rare and precious local species in Tianzhu Tibetan Autonomous County, China. However, the number of white yak is decreasing due to their low rate of reproduction [1]. Therefore, a number of recent studies focused on its reproduction and physiology [2–4], as well as assisted reproductive techniques [1,5]. Zhang *et al.* (2006) investigated the breeding conditions of female yaks at the plateau. Female yaks calving a calf biennially, triennially and annually accounted respectively for 88.84%, 9.51% and 1.65% of the total reproductive females in the heard [6].

During follicular development, the primordial follicle develops into the primary follicle, and then into the secondary follicle. The primordial, primary and secondary follicles are also called the pre-antral follicle because they are all cavum-absent. The cavity then gradually appears and grows bigger as the secondary follicle grows, going from irregular- to crescent-shaped and becomes filled with follicular fluid. As the cavity forms, the oocyte, surrounded by several layers of granular cells, is squeezed to one side of the cavity and formed the cumulus oophorus. The ovarian follicle further grows into a mature follicle, after which, together with the follicular fluid, the oocyte is expelled as ovulation proceeds. In each estrous cycle, there are also two to three other waves of follicular development in which several oocytes develop to tertiary follicles; however, usually only one oocyte, sometimes more, develops to the size of a mature follicle, and the others would undergo follicular atresia.

It is well established that early antral follicular development in cattle is not gonadotropin-dependent, and the follicle stimulating hormone (FSH) receptor is expressed even on bovine follicles less than 2 mm in diameter [7]; however, follicles that are 4–5 mm in diameter are recruited by the wave of follicular growth [8]. Oocytes in follicles less than 2 mm in diameter cannot undergo spontaneous nuclear maturation [9] but can grow and attain meiotic capability while the follicle is 2–3 mm in diameter [10]. Furthermore, the status of oocyte development can be assessed by the size of the follicle [9]. Therefore, follicles of Tianzhu white yak were divided into four groups according to their follicular diameter: 0–2, 2–4, 4–6 mm and greater than (>) 6 mm.

Follicular fluid proteins originate from the secretory activity of the surrounding theca cells and granulosa, and from the transfer of blood plasma components that cross the blood–follicle barrier [11]. Follicular fluid contains growth factors, members of the transforming growth factor-beta (TGF-β) superfamily, interleukins, reactive oxygen species (ROS), anti-apoptotic factors, proteins, sugars, and prostanoids. These substances are related to the metabolic activity of ovarian cells and reflect the physiological status of follicles [12]. Furthermore, follicular fluid can be used to assess oocyte quality, determine the subsequent potential of achieving fertilization and embryo development, as well as understand the mechanisms of follicular differentiation and development [11,13].

The proteome, as a complex and dynamic entity, can provide a rich and varied source of data. Proteomics is a method of high-flux and systematic analysis of proteins. Thus far, proteomic analyses of follicular fluid have been performed for human, bovine, porcine, canine, and mare species. The variability of the proteome in the follicular fluid of these species during the follicular development was established; however, no protein was found to be differentially expressed in follicular fluid during the early dominant, late dominant, and pre-ovulatory stages of mare follicular development [12]. In this investigation, a proteomics study of white yak follicular fluid detected changes in the expression of follicular fluid proteins at different stages of follicular development and provided the basis for improving the fertility of white yak.

## 2. Results and Discussion

### 2.1. Analysis of Differentially Expressed Protein Spots

**Figure 1.**
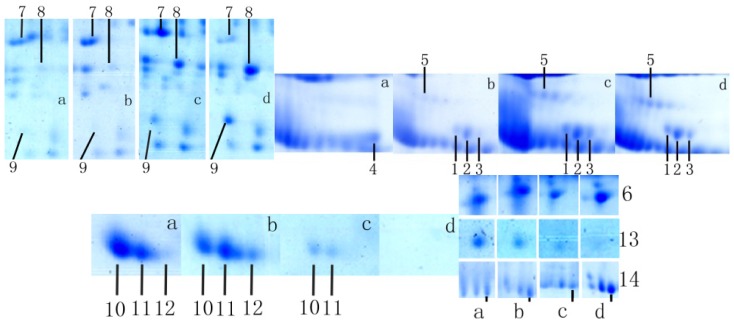
Two-dimensional gel electrophoresis maps of follicular fluid in Tianzhu white yak. Follicle diameter: (**a**) 0–2 mm; (**b**) 2–4 mm; (**c**) 4–6 mm; (**d**) >6 mm. 1–14 indicate the identified proteins.

A two-dimensional gel electrophoresis (2-DE) map was generated from the proteins expressed in Tianzhu white yak follicular fluid during diestrus (Figure 1). Upon comparison of the protein expression levels in the follicular fluid of the four studied groups, a total of 34 proteins were determined to be differentially expressed, as calculated by PDQuest software.

### 2.2. Identification and Analysis of Differentially Expressed Proteins

After comparing the generated 2-DE map with the 2-DE maps of previous studies of follicular fluid in different species (human, bovine, porcine, canine and mare), we rejected some spots considered indicative of potentially highly abundant proteins and hardly excised spots. Thereafter, 14 spots of significant difference remained, and these were selected for analysis by matrix-assisted laser desorption/ionization time of flight mass spectrometry (MALDI-TOF/TOF-MS). The 14 selected spots were identified as unique proteins, 10 of which were validated (protein score ≥60). The proteins AS3MT, ANKRD 6, MRP4-like, MAPKAP1, AGO3, AGP, AR, OBSL1, RNF20, crystalline profilin-beta-actin, protein SPT2 and protein C10orf107 were reported for the first time in follicular fluid.

**Figure 2.**
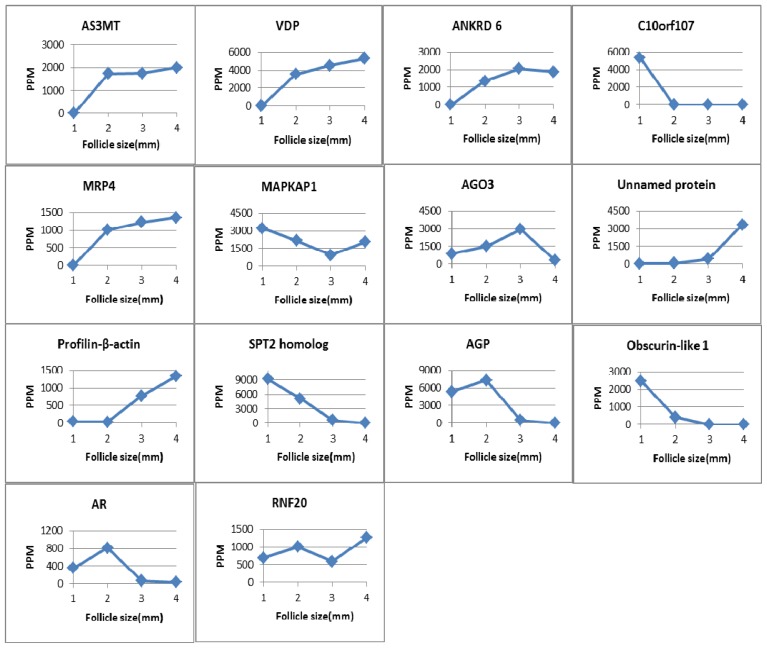
The contents of the differentially expressed proteins in the four ovarian follicle groups (1,2,3,4) correspond to follicle size: 0–2, 2–4, 4–6, and >6 mm, respectively. PPM calculated by PDQuest software.

AS3MT, MRP4-like, VDP and ANKRD 6 were not detected in yak follicles less than 2 mm in diameter; protein C10orf107 was expressed only in follicles less than 2 mm in diameter, while protein SPT2 homolog and AGP were not detected in follicles >6 mm and obscurin-like-1 was expressed in follicles less than 6 mm. The other proteins were expressed during the process of follicular development. The features of the 14 proteins are summarized in Table 1. The changes in the level of expression of the 14 identified differentially expressed proteins are summarized in Figure 2.

#### 2.2.1. Arsenic (+3) Methyltransferase

Arsenic, as a naturally existing element, shows potent toxic and mutagenic effects, but has been reported to possibly play an essential role in human health [14]. Arsenic (+3) methyltransferase (AS3MT) catalyzes the formation of mono-, di-, and tri-methylated metabolites from inorganic arsenic in the +3 oxidation state. Regular oral administration of arsenic to mature female albino rats resulted in the down-regulation of downstream components of the estrogen signaling pathway and the inhibition of gonadotropin and estradiol generation [15]. The mRNAs of *Cyp17*, *3-HSD*, *P450scc*, and testicular testosterone synthesis enzyme were significantly decreased in As_2_O_3_ (Arsenic trioxide)-treated male mice [16]. The enzymes are all related to the biosynthesis of androgen, estrogen and testosterone in ovarian theca cells, follicular development, and atresia [8]. *3β-HSD* and *P450scc* mRNAs were not expressed in bovine follicular granulosa cells of pre-antral and early antral follicles (<4 mm) [8]. As ovarian and follicle diameter are smaller in Tianzhu white yak than in cattle [10,17,18], AS3MT was not expressed in follicles <2 mm in this study. We conjecture that *P450scc* and *3β-HSD* would be expressed in yak follicles with a diameter greater than 2 mm; thus, AS3MT as an endogenous mammalian substance, is expressed after the expression of P*450scc* and *3β-HSD*. At follicular diameters >2 mm, the levels of AS3MT increased as the follicular diameter increased. AS3MT would down-regulate testicular testosterone synthesis by repressed the expression of P450scc and 3β-HSD protein.

#### 2.2.2. Vitamin D-Binding Protein

Vitamin D-binding protein (VDP) is responsible for the stepwise activation of vitamin D_3_ to 25(OH)D_3_ and 1,25(OH)_2_D_3_, and for transporting these small molecules to the organs and cells in which they are required. In follicular fluid, higher vitamin D levels corresponded to lower glucose concentrations, and low or high glucose concentrations were harmful to the development of the granule cells as well as to oocyte maturation [19]. Estes *et al.* [20] reported that, in patients undergoing *in vitro* fertilization(IVF), IVF was prone to success when VDP level of follicular fluid is between 0.43 and 20 ng/mL [21], but had a negative effects on embryo quality at a high VDP concentration (>20 ng/mL) in follicular fluid [19]. In healthy women, the concentrations of 25(OH)D_3_ and 1,25(OH)_2_D_3_ remain constant in the follicular fluid during gonadotrophin-induced ovarian stimulation [22], and the serum VDP levels do not change significantly [23]. Therefore, appropriate VDP levels are necessary for oocyte maturation in the follicle. However, we did not detect VDP expression in Tianzhu white yak follicles with a diameter less than 2 mm, and the VDP levels increased with increase of follicular diameter. In cattle, oocyte maturation only occurs in follicles between 2 and 3 mm in diameter [10]. Vitamin D also functions in regulating the expression of the aromatase gene in estrogen biosynthesis [24]. 1,25(OH)_2_D_3_ also decreases androstenedione production in human theca cells [25]. Furthermore, vitamin D is associated with follicular atresia; therefore, VDP may play a role during the process of folliculogenesis.

#### 2.2.3. Mitogen Activated Protein Kinase 2-Associated Protein-1

Rictor-mTOR (TORC2), a part of mammalian target of rapamycin (mTOR), is known to control cell proliferation and growth [26]. Mitogen activated protein kinase 2-associated protein-1 (MAPKAP1) is a component of TORC2 [27]. TORC2 activates Akt which is required for maintaining cellular size and morphogenesis within the follicular epithelium in *Drosophila* [28]. Inhibition of mTOR in ovarian follicles results in compromised granulosa proliferation, reduces the number of eggs ovulated, and hampers follicle growth, suggesting that mTOR may be associated with selection of the dominant follicle [29]. Moreover, a group of follicles 4–5 mm in diameter would be recruited, even though nearly 90% of follicles are atretic during the process of development in normal ovarian. In this study, the levels of MAPKAP1 were lowest in follicles of a diameter 4–6 mm, which indicates that MAPKAP1 may also relate to follicular development, oocyte maturation, and selection of the dominant follicle.

#### 2.2.4. Argonaute-3

Argonaute-3 (AGO3) is a member of the Ago subfamily of proteins, which functions to guide gene silencing after transcription. AGO3 is a major component of the RNA-induced gene silencing complex (RICS). An earlier study in *Drosophila melanogaster* found that AGO3 silences transposons by circularly recombining with the Piwi and Aubergine proteins to amplifiy piRNA [30]. piRNA is a new pathway of cell development [31], while Aubergine is also associated with oogenesis [32]. AGO3 has been found to be expressed in germ line cells of the egg chamber and cap cells of the germarium in *Drosophila* [33], and mutation of AGO3 leads to polar fracture of oocytes [34]. In the current study, the levels of AGO3 were highest in follicles of 4–6 mm diameter, indicating that AGO3 functions not only in oocyte development, but also plays a significant role in the development of follicles in the Tianzhu white yak.

#### 2.2.5. Aldose Reductase (AR)

The major function of aldose reductase (AR) is to mediate the metabolism of glucose via the polyol pathway. As previously mentioned in Section 2.2.2, glucose levels are associated with the growth of oocytes and granulosa cells. In diabetic mice, AR suppressed the maturation of meiotic oocytes by metabolizing glucose in the polyol pathway [35]. In macrophages, inhibition of AR prevents the expression of inducible nitric oxide synthase [19] and the production of nitric oxide (NO) [36]. NO plays a vital role in follicular development, oocyte maturation and ovulation, luteinization, and degeneration of the corpus luteum [7]. In a study of rats, Iwata *et al*. [37] reported that, from the early stage of proestrus to early stage of estrus, AR levels changed from maximum to minimum, indicating that AR is regulated by gonadotropic hormone E3 ubiquitin-protein ligase BRE1A (RNF20) is an E3 ligase for histones H2B monoubiquitination during the murine estrous cycle. In bovine oocytes, AR is ubiquitously expressed throughout the maturation process (GV, M I, M II); however, phosphorylation of the enzyme increases first and then dramatically decreases in the M II stage [38]. When considering the follicle size and oocyte maturation of Tianzhu white yaks, we observed that the expression of AR in follicular fluid during early follicular development is consistent with the levels of AR phosphorylation during oocyte maturation. Therefore, AR is regulated by gonadotropic hormonee during the estrous cycle and associated with oocyte development and follicular development.

#### 2.2.6. E3 Ubiquitin-Protein Ligase BRE1A (RNF20)

E3 ubiquitin-protein ligase BRE1A (RNF20) is an E3 ligase for histones H2B monoubiquitination. RNF20 was first found to be expressed in the human testis [39]. In prostate cancer cells, both RNF20 and RNF40 are able to modulate the transcriptional activity of the androgen receptor [39], which is related to folliculogenesis [40]. In this study, the levels of RNF20 were lowest in follicles 4–6 mm in diameter. Therefore, RNF20 may relate to the development of follicles in Tianzhu white yak.

## 3. Materials and Methods

### 3.1. Collection of Follicular Fluid

Thirty ovaries in total were collected from 15 healthy, not-pregnant white yak (aged 3–4 years, 3–4 months after estrus) in a local abattoir immediately after slaughter, placed in a thermos containing physiological saline (with penicillin and streptomycin) at 37 °C, and taken to the laboratory within 12 h. The follicles were divided into four groups according to follicular diameter: 0–2, 2–4, 4–6, and >6 mm. The follicular fluid was directly aspirated from the follicles and mixed according to size, then centrifuged at 1500× *g* for 10 min to remove cells and other debris. The fluid supernatants were observed under an inverted microscope (IX-71, Olympus, Tokyo, Japan) to determine whether any oocytes were present, and then frozen at −80 °C until further analysis.

### 3.2. Two-Dimensional Gel Electrophoresis

Two-dimensional gel electrophoresis (2-DE) was performed according to the manufacturer’s instructions (Bio-Rad; Hercules, CA, USA). Protein quantity was determined by a modified Bradford method [41]. The follicular fluid samples were thawed at room temperature and diluted with hydration solution (0.5% (*v*/*v*) IPG buffer, 0.001% bromophenol blue, 7 M urea, 2 M thiourea, 65 mM dl-Dithiothreitol (DTT) and 4% 3-[(3-cholamidopropyl) dimethylamonio]-1-propane sulfonate), then centrifuged at 10,000× *g* for 5 min at 4 °C. The protein was draped with a 17 cm IPG strip (pH 3–10 NL, Bio-Rad) after being placed in the hydration tray for isoelectric focusing with a PROTEAN IEF Cells (Bio-Rad). A standard procedure was used: rehydration for 14 h [42] at 50 V, with focusing in four steps: 250 V for 1 h, 1000 V for 1 h, 9000 V for 6 h, and 9000 V for 80,000 h.

After focusing, the gels were incubated with 5 mL equilibration buffer (0.375 mol/L Tris, pH 8.8, 130 mmol/L DTT, 6 mol/L urea, 20% glycerol, 2% SDS) for 15 min, then equilibrated in another equilibration buffer (2.5% iodoacetamide, 20% glycerol, 2% SDS, 0.375 mol/L Tris, pH 8.8, 6 mol/L urea) for 15 min. After rinsing, the gels were transferred to a 12% gel for SDS-PAGE, which was also conducted in the PROTEAN II xi 2DE Cells (Bio-Rad) at 50 V for 1 h, and at 200 V to finish.

### 3.3. Staining and Image Analysis

Gels were stained with Coomassie Brilliant Blue G-250 overnight, rinsed with deionized water, and scanned using a PowerLook 2100XL scanner (UMAX, Xinzhu, Taiwan). The gel images were analyzed by PDQuest 8.0 software (Bio-Rad).

### 3.4. Peptide Identification

Spots of interest were manually excised, destained, digested with trypsin, and analyzed by MALDI-TOF/TOF-MS. Spots were destained in 30 mM potassium ferricyanide/100 mM sodium thiosulfate (1:1 (*v*/*v*)) for 20 min, incubated in 0.2 M NH_4_HCO_3_ for another 20 min, and then lyophilized. Each spot was digested in 25 mM NH_4_HCO_3_ and 12.5 ng/mL trypsin overnight. The peptides were extracted three times with 60% acetonitrile (ACN)/0.1% trifluoroacetic acid (TFA), and then pooled and dried. Sequencing analysis was performed by Beijing Protein Innovation (Beijing, China) ULTRAFLEX × 2TOFTOF. The peptides were identified using MASCOT software (Matrix Science: Boston, MA, USA) by searching against the NCBI database. If the protein scored above 60, then it was validated (*p* < 0.05).

### 3.5. Statistical Analysis

All results were presented as mean ± SD. The Mann–Whitney nonparametric test was used to analyze MASCOT Score. One-way analysis of variance (ANOVA) was used to analyze results. Significant differences were defined as *p* < 0.05, and were determined using statistical software package SPSS 11.0 (Version 11.0; Chicago, IL, USA).

## 4. Conclusions

In summary, this study identified 14 differentially expressed proteins in the follicular fluid of Tianzhu white yak during diestrus. Some of these differentially expressed proteins are related to the carbohydrate metabolism, sex hormone synthesis, signal transduction, cell developmental pathways, and cytoskeletal rearrangement that occurs during the processes of oocyte development and folliculogenesis. However, the functions of these proteins in reproduction require further verification. This study provides a solid basis for further research into the processes that regulate follicular development and oocyte maturation in Tianzhu white yak.

## Figures and Tables

**Table 1.** Matrix-assisted laser desorption/ionization time of flight mass spectrometry (MALDI-TOF/TOF-MS) identification of differentially expressed proteins in the follicular fluid of Tianzhu white yak.

No.	(NCBI) Accession	Protein Name	PI/MW (kDa)	Peptide No.	Coverage (%)	MASCOT Score
1	gi|78042514	Arsenic (+3) methyltransferase *[Bos taurus]*	5.36/42.51	7	26	65
2	gi|85701291	Vitamin D-binding protein *[Bos taurus]*	5.36/54.90	16	34	102
3	NP_001192406.1	Ankyrin repeat domain-containing protein 6 *[Bos taurus]*	9.42/80.17	4	7	35
4	gi|157278953	C10orf107 protein *[Bos taurus]*	6.92/21.19	5	4	50
5	XP_001256333.2	Multidrug resistance-associated protein 4-like *[Bos taurus]*	9.53/22.29	3	20	35
6	gi|187611460	Mitogen activated protein kinase 2-associated protein-1	7.24/59.54	4	12	66
7	gi|229544700	Protein argonaute-3 *[Bos taurus]*	9.22/98.38	8	15	66
8	gi|21538351	Unnamed protein *[Bos taurus]*	6.36/34.24	11	41	79
9	gi|157881403	Crystalline profilin-beta-actin *[Bos taurus]*	5.29/41.92	9	29	63
10	gi|329664588	Protein SPT2 homolog *[Bos taurus]*	9.79/75.44	8	11	48
11	gi|121957959	Alpha-1-acid glycoprotein	5.62/23.45	7	35	65
12	gi|111305443	Obscurin-like-1 *[Bos taurus]*	5.15/68.96	7	13	67
13	gi|162652	Aldose reductase (EC 1.1.1.21), partial *[Bos taurus]*	5.68/34.35	6	19	72
14	gi|182627578	E3 ubiquitin-protein ligase BRE1A *[Bos taurus]*	5.70/114.27	11	14	64
